# Advanced atomic force microscopy techniques III

**DOI:** 10.3762/bjnano.7.98

**Published:** 2016-07-21

**Authors:** Thilo Glatzel, Thomas Schimmel

**Affiliations:** 1 Department of Physics, University of Basel, Klingelbergstrasse 82, 4056 Basel, Switzerland; 2 Institute of Nanotechnology (INT), Karlsruhe Institute of Technology (KIT), 76021 Karlsruhe, Germany

**Keywords:** atomic force microscopy

Atomic force microscopy (AFM) celebrates its 30th anniversary this year. It was presented by Binnig, Quate and Gerber in 1986 as an extension of the scanning tunneling microscope (STM) with the possibility to measure forces as small as 10^−18^ N [1]. Since then many different variations of the force detection method and various applications have appeared [2–3] and still the scientific community is inventing advanced and improved detection mechanisms and fields of operation. The limits of resolution are not reached yet, however, some research groups start characterizing even ”bond-like” features in high-resolution measurements in between atoms of single molecules or molecular assemblies at positions where chemical bonds are expected [4–6]. However, the origin of this contrast is under intense discussion [7–8]. The extension of the technique towards other physical properties in surface science is pushed continuously. Combined AFM and STM measurements reveal related force and electronic properties [9], energy dissipation in manipulation processes can be examined via the excitation voltage needed to keep a constant amplitude of the probe oscillation [10–11], pulling forces of atomic or molecular wires can be determined and compared with theoretical predictions [12–13], and local charges within single molecules can be measured [14–15], however, the quantification is still under intense discussion. Chemical reactions are triggered and imaged by the AFM tip [16–17] and of course the technique is not limited to ultrahigh-vacuum (UHV) and low-temperature conditions. Therefore, the traditional field in surface science based on diffraction and scattering of charged particles, mostly electrons, which are used as probes in a variety of experimental methods is extended by a powerful local and real space imaging and characterization technique.

This third Thematic Series of „advanced atomic force microscopy techniques“ assembles 22 exciting articles around the improvement and application of this technique which appeared in the *Beilstein Journal of Nanotechnology* within the past 1.5 years. The articles can roughly be grouped in two major categories: local measurements of mechanical properties and high resolution force measurements and spectroscopy.

The characterization of mechanical properties by AFM manifests themselve primarily in the local detection of adhesion, friction and elasticity. Georg Fantner and his co-workers developed an advanced microscope capable of obtaining nanoscale topography as well as mechanical properties by multifrequency AFM at high speed. They combined recent progress in increased imaging speed and photothermal actuation in a unique and versatile AFM head using ultrasmall cantilevers [18]. Single-cell force spectroscopy is used by a biophysics group around Jonne Helenius to quantify the contribution of cell adhesion to specific substrates at both the cell and single molecule level [19]. Furthermore, physico-mechanical properties of intestinal cells were elucidated by force curve measurements by Eva Roblegg and co-workers [20]. The local elastic stiffness and damping of individual phases in a titanium alloys was measured by using atomic force acoustic microscopy (AFAM) and mapping of contact-resonance spectra [21]. Another alloy, namely a Pt containing metallic glass, was characterized by AFM indentation in UHV to quantitatively determine the hardness and deformation mechanisms by Arnaud Caron and Roland Bennewitz [22]. Santiago Solares and Enrique A. López-Guerra presented different approaches to model such viscoelastic properties within AFM simulations [23]. Sliding contact properties like several transitions in the friction coefficient with increasing load have been found on Au(111) in sulfuric acid electrolyte containing Cu ions by Helmut Baltruschat an co-workers [24] and the stiffness of micron-sized sphere-plate contacts was studied by Diethelm Johannsmann et al. by employing high frequency tangential excitation [25].

On the other hand, force spectroscopy and advanced imaging and analysis techniques form a major part of this Thematic Series. For all AFM experiments the tip condition is one of the most critical parameters, Fei Long et al. presented a method for single-molecule probe modification by using a Cu-catalyzed alkyne–azide cycloaddition reaction [26]. To quantify the performed measurements in a reproducible way is still a challenging topic in all AFM measurements. In amplitude modulation AFM higher harmonic signals can be used, Kfir Kuchuk and Uri Sivan presented a mathematical model to derive an accurate and explicit formulae for both conservative and dissipative forces in terms of an arbitrary single harmonic [27], while Luca Costa and Mario S. Rodrigues derived formulas for the tip–sample interactions and investigated the effect of spurious resonances on the measured interaction in liquid media [28]. A software package, dForce, was also presented that allows for a better understanding of amplitude modulation and bimodal AFM experiments in air or liquid [29]. A method for the calibration of the torsional force by using human fibronectin and its monoclonal antibody was presented by Andrzej J. Kulik and co-workers [30]. High-resolution measurements of the adhesion effect of a water film on CaF_2_ [31], electric and transport phenomena determined by liquid KPFM in ionically-active and -inactive liquids [32], the spray deposition of single molecules to insulating and ionic surfaces [33], and combined STM and AFM measurements on single-layer graphene on SiC(0001) [34] have been investigated, discussed, and presented. Another combined STM-AFM study determines very accurately the probe-nanocrystal interaction potential [35]. Finally, enhanced information can also be achieved by more accuratly adjust the used parameters and intensivly analyse the results [36–38].

As detailed above this Thematic Series “Advanced atomic force microscopy techniques III” sums up fundamental and applied progresses in surfaces science with a clear focus on advanced atomic force microscopy. It continues the Thematic Series “Advanced atomic force microscopy techniques I and II” [39–40]. The articles demonstrate again, that despite the already 30th anniversary of AFM, current developments in this research field are impressive and by far not finished and we hope to stimulate also further research groups to take part and apply these successful techniques to many other exciting surfaces, systems and materials.

Finally, we want to thank all authors for contributing their excellent work to this series and especially the referees for their continuous support by providing sound and objective reports. Here, we also want to acknowledge the more and more important Open Access policy of the *Beilstein Journal of Nanotechnology* providing the possibility to continue this exciting and stimulating Thematic Series freely accessible worldwide. Moreover, we would like to thank the editing team of the Beilstein-Institut for the excellent and professional framework and support allowing for the collection, review, publishing, and distribution of research results in an easy and excellent way.

Thilo Glatzel and Thomas Schimmel

Basel and Karlsruhe, June 2016
